# Investigating the effects of combined treatment of mesalazine with *Lactobacillus casei* in the experimental model of ulcerative colitis

**DOI:** 10.3389/fmolb.2024.1456053

**Published:** 2024-10-03

**Authors:** Shabnam Bahrami, Nahid Babaei, Hadi Esmaeili Gouvarchin Ghaleh, Jaleh Mohajeri Borazjani, Mahdieh Farzanehpour

**Affiliations:** ^1^ Department of Molecular Cell Biology and Genetics, Bushehr Branch, Islamic Azad University, Bushehr, Iran; ^2^ Applied Virology Research Center, Biomedicine Technologies Institute, Baqiyatallah University of Medical Sciences, Tehran, Iran; ^3^ Department of Fisheries and Natural Resources, Bushehr Branch, Islamic Azad University, Bushehr, Iran

**Keywords:** inflammatory cytokine, inflammation, *Lactobacillus* casei, mesalazine, probiotic, ulcerative colitis

## Abstract

**Introduction:**

Ulcerative colitis (UC), a common gastrointestinal disorder in affluent nations, involves chronic intestinal mucosal inflammation. This research investigated the effects of combined probiotic treatment of *Lactobacillus casei* (L. casei) and mesalazine on disease activity index and inflammatory factors in the UC model.

**Methods:**

20 male BALB/c mice were utilized and divided into four groups. To induce UC, all groups received 100 μL of 4% acetic acid (AA) intra-rectally. The first group received phosphate-buffered saline (PBS) (as a control group), the second group was treated with L. casei, the third group was treated with mesalazine and, the fourth group was treated with L. casei and mesalazine. Treatment with L. Casei and mesalazine commenced after the manifestation of symptoms resulting from UC induction. Finally, the mice were euthanized and the disease activity index, myeloperoxidase activity, nitric oxide rate, cytokines level (IL-1β, IL-6, TNF-α) and, gene expression (iNOS, COX-2, and cytokines) were evaluated.

**Results:**

The combined treatment of L. casei and mesalazine led to a significant decrease in the levels of NO, MPO and inflammatory cytokines. In addition, the expression of cytokines, iNOS and COX-2 genes decreased in mice treated with the combination.

**Discussion:**

This study shows that combined treatment of L. casei and mesalazine improves of experimental UC, which can be attributed to the anti-inflammatory properties of L. casei and mesalazine. In conclusion, this combination therapy can be considered a suitable option for the management of UC.

## Introduction

Ulcerative colitis (UC), a recurrent and ongoing form of inflammatory bowel disease (IBD), has unique characteristics related to inflammation and localization. UC typically commences in the rectum and remains confined to the colon (Makević et al., 2023). IBD has a complex etiology involving disruptions in physiology, microbiology, immunology, and genetics (Bermudez-Brito et al., 2012). The intestinal bacterial flora, mucosal immune dysregulation, and genetic susceptibility contribute to the development of IBD, resulting in various signs consisting of abdominal pain, diarrhea, and rectal bleeding. Additionally, this condition can also cause extra-intestinal manifestations (Ohlsson, 2022). The best treatments for UC involve using corticosteroids, aminosalicylates, and immunosuppressants. However, the severe side effects of these drugs limit their long-term use. Some medications may lead to severe adverse effects, including gastrointestinal disturbances, pancreatitis, liver and kidney disorders, and gastrointestinal ulcers. Mesalazine, also known as aminosalicylic acid, is the first-line treatment for IBD and is the primary treatment for mild to moderate UC. It works by inhibiting lipoxygenase and cyclooxygenase intermediates, as well as IL-1, IL-2, and tumor necrosis factor-α, effectively controlling active inflammation and promoting intestinal tissue healing (Iacucci et al., 2010). However, many patients do not respond well to this treatment. If inflammation is not controlled, individuals with UC are at risk of developing colorectal cancer (Bohm et al., 2020). Extensive research focuses on modulating beneficial microbes to alter the balance of gut bacteria, reduce inflammation, and prevent relapses in UC (Cristofori et al., 2021). Probiotics, notably *Lactobacillus* species, are a pivotal constituent of the intestinal microflora (Qin et al., 2022). An imbalance in gut microorganisms and improper recognition of commensal bacteria by the immune system have been associated with illnesses such as IBD (Santana et al., 2022). Specific probiotic bacteria have shown potential to provoke protective anti-inflammatory responses and reduce colitis development in animal models, demonstrating promise for managing chronic UC in humans (Guo and Lv, 2023). Among these, L. casei, a commensal bacterium, is naturally present in the gut of healthy individuals (Xu et al., 2020). Recent investigations have revealed the efficacy of certain probiotics in improving lifestyles for IBD cases, highlighting the significance of the human intestinal microbiota and its functions, including protection against pathogenic microbes and regulation of epithelial innate immunity (Roy and Dhaneshwar, 2023). Dysbiosis is commonly associated with IBD, underlining the influence of microbial balance or imbalance in disease pathogenesis (Stolfi et al., 2022). Evidence indicates that the presence of commensal enteric bacteria and their byproducts influences the progression of IBD, underscoring the potential of probiotics to enhance intestinal diversity and alleviate symptoms in individuals with IBD (Štofilová et al., 2022). These probiotics, capable of suppressing inflammation or activating innate immunity, present therapeutic potential in restoring the host’s gut microbiota (Kaur and Ali, 2022). Moreover, probiotic microorganisms generate lactic acid and lactate, which can impede the growth of detrimental organisms and exhibit an anti-inflammatory impact on the gastrointestinal tract (Sun et al., 2021). Pathogenic bacteria can activate TLR4, which can cause a disruption in the gut barrier and lead to allergen translocation within the intestinal mucosa. As a result, pro-inflammatory cytokines such as TNF-α, IL-1ß, and IL-6 may be produced (Kaur and Ali, 2022). The progressive nature of this disease and the costs associated with its treatments pose a growing economic burden on health systems globally (Zhao et al., 2021). This research aims to investigate the effects of combined treatment with L. casei and mesalazine on several aspects of inflammation in the UC model.

## Materials and methods

### Experimental model

The study utilized male BALB/c mice, aged 6–8 weeks and weighing between 25 and 30 g, obtained from the Baqiyatullah University of Medical Sciences (BMSU) animal care facility. These mice were housed in controlled environments with a regular day-night cycle, a temperature of 23°C ± 2°C, and appropriate humidity levels. They had unrestricted access to food and water. The study procedure was approved by the Baqiyatullah University of Medical Sciences Research Ethics Committee (Approval ID: IR.BMSU.AEC.1400.001).

### *Lactobacillus casei* culture

The BMSU provided a probiotic species L. casei ATCC: 393, which was cultivated on Man- Rogosa-Sharpe agar and kept anaerobically at 37°C for 72 h. After adjusting the probiotic strain’s concentration of 10^9^ CFU, PBS was used two times for washing.

### Acetic acid-induced ulcerative colitis

In the process of inducing UC, before the intrarectally administration of AA, the mice were provided unrestricted access to water while undergoing a 36-h fasting period. Following the fasting period, 100 µL of a 4% AA solution was injected into the rectum several times. After the injection, the mice were maintained in a slantwise position for 30 s. Ten days after the last injection and following the onset of symptoms (Diarrhea and bloody stools) of the disease, the treatment began. The research involved 20 male BALB/c mice that were randomly allocated into four equal groups: control group (PBS, orally, daily), L. casei (10^9^ CFU, orally, daily), mesalazine (30 mg/mL, orally, daily), and L. casei + Mesalazine (10^9^ CFU + 30 mg/kg, orally, daily) in 100 µL PBS (Table 1) (Gheibi et al., 2018). After 1 month of treatment, the mice were euthanized, and the intestine and spleen tissues were isolated for analysis (Herias et al., 2005).

**TABLE 1:** Features of the groups that were studied.

*Group*	*Abbreviations*	*Characteristics (in 100* *μL of PBS)*
*control group*	C.G	PBS
*L. casei*	L	10^9^ CFU, orally, daily
*Mesalazine*	M	30 mg/kg, orally, daily
*L. casei + Mesalazine*	L + M	10^9^ CFU + 30 mg/kg, orally, daily

### Homogenization of intestine tissue

The mice were euthanized using ketamine (100 mg/kg) and xylazine (10 mg/kg). Approximately 2 cm of distal intestine tissue was excised, opened, and washed with PBS to remove feces and visible fat. Then it was homogenized in a saline solution that was ten times more in volume and kept at a very low temperature. This mixture was further centrifuged at 10,000 × g at 4°C for 10 min (Mashhouri et al., 2020).

### Disease activity index (DAI)

To calculate DAI in mice, stool consistency and blood in stool severity were recorded and the individual scores for each parameter provided a quantitative measure of the severity of colitis (Table 2) (Mashhouri et al., 2020).

**TABLE 2:** DAI scoring system.

*Consistency of stool*	*Blood in Stool*	*Score*
*Normal*	Normal	0
*Soft*	Red	1
*Very soft*	Dark Red	2
*Diarrhea*	Black	3

### Macroscopic and microscopic assessments

Colon tissue samples were taken and histological evaluations were performed. 10% formalin solution was used to fix the samples. After sectioning, they were stained with hematoxylin and eosin (H&E). Scoring for measurements was done by an impartial observer (Table 3). Histopathological evaluations were scored on a scale from 0 to 4. Scores for each parameter were summed to determine the overall severity of tissue damage (Gheibi et al., 2018).

**TABLE 3:** Assessing intestinal mucosal condition and scoring methodology.

*score*	Characteristics
*0*	No inflammation
*1*	Redness of the mucous membrane
*2*	Mild edema, bleeding and erosion
*3*	Edema, bleeding and moderate ulceration
*4*	Edema, severe bleeding and tissue necrosis

### Measuring MPO assessment

MPO is often used as a biochemical marker to determine the presence of granulocytes, specifically neutrophil infiltration in gastrointestinal tissues. To measure the MPO activity level, a previously described method was adopted (Mashhouri et al., 2020). The sample was homogenized and mixed with H_2_O_2_ and TMB solution. The mixture was then assessed for absorbance at 450 nm with a reference of 620 nm on a microplate reader. After incubating the samples at 37°C for 15 min, the reaction was stopped using H_2_SO_4_ and absorbance was measured again. The MPO activity was determined based on the difference in absorbance concerning the standard curve of HRP. The results were presented as millunits per milliliter (mU/mL).

### NO activity assessment

To quantify the NO concentration within colonic tissues, the Griess reagent, a commonly utilized colorimetric method, was employed. Specifically, 50 µL of Griess reagent (composed of 0.1 per cent sulfanilamide, 3 per cent phosphoric acid, and 0.1 per cent naphthyl ethylenediamine) was mixed with an equal amount of homogenized colonic tissue. This mixture underwent incubation in darkness at 25°C for 10 min. Subsequently, the amount of absorbance at 540 nm was measured. Analysis of nitric oxide concentration in colon tissue samples involved correlating absorbance values with the sodium nitrite standard curve (Mashhouri et al., 2020).

### Cytokines assay

To quantify the levels of IL-1β, TNF-α, and IL-6 ELISA kits from the Peprotech Company were used. The evaluation of culture supernatants followed the instructions and guidelines provided by the manufacturer (Jahantigh et al., 2023).

### Inflammatory genes and cytokines expression

Using the Pars Tous kit and the manufacturer’s instructions, colon tissue was subjected to RNA extraction. Subsequently, cDNA synthesis was conducted. To amplify the IL-1β, IL-6, COX-2, TNF-α, and iNOS genes in mice, unique primer sequences for the forward and reverse were designed utilizing Oligo7 primer design software and validated via the NCBI website. The housekeeping gene Glyceraldehyde-3-Phosphate Dehydrogenase (GAPDH) was utilized with the primer set outlined in Table 4. The RT-PCR procedure consisted of forty Polymerase Chain Reaction cycles after a 30-s initial denaturing step at 95°C (Tehranipour et al., 2018).

**TABLE 4:** The sequence of primers.

Gene	Primers
*COX-2* Forward primer Reverse primer	5′CTT CGG GAG CAC AAC AGA GT3′ 5′AAG TGG TAA CCG CTC AGG TG3′
*iNOS* Forward primer Reverse primer	5′CAC CTT GGA GTT CAC CCA GT3′ 5′ACC ACT CGT ACT TGG GAT GC3′
*IL-1ß* Forward primer Reverse primer	5′GCC ACC TTT TGA CAG TGA TGAG3′ 5′AAG GTC CAC GGG AAA GAC AC3′
*IL-6* Forward primer Reverse primer	5′CAA CGA TGA TGC ACT TGC AGA3′ 5′TGT GAC TCC AGC TTA TCT CTTGG3′
*TNF-α* Forward primer Reverse primer	5′AGG CAC TCC CCC AAA AGA TG3′ 5′CCA CTT GGT GGT TTG TGA GTG3′
*GAPDH* Forward primer Reverse primer	5′GGG TCC CAG CTT AGG TTC AT3′ 5′CAT TCT CGG CCT TGA CTG TG3′

### Statistics analysis

The statistical data were presented as Mean ± SEM. The Kruskal–Wallis test was used to compare score differences among various groups. In the case of non-parametric values (discontinuous ranks associated with disease severity), the Mann-Whitney U test with Bonferroni correction was employed. For continuous data, a one-way analysis of variance (ANOVA) was conducted along with Dunnett’s *post hoc* test after confirming their normal distribution through the Kolmogorov-Smirnov test. Any significance level below *p* < 0.05 was considered statistically significant. Molecular data analyses were done using REST (2009) software, and other analyses were performed using SPSS (ver. 24). All graphs were created using Graph Pad Prism (ver. 8) software.

## Results

### Disease activity index and microscopic assay

**FIGURE 1:**
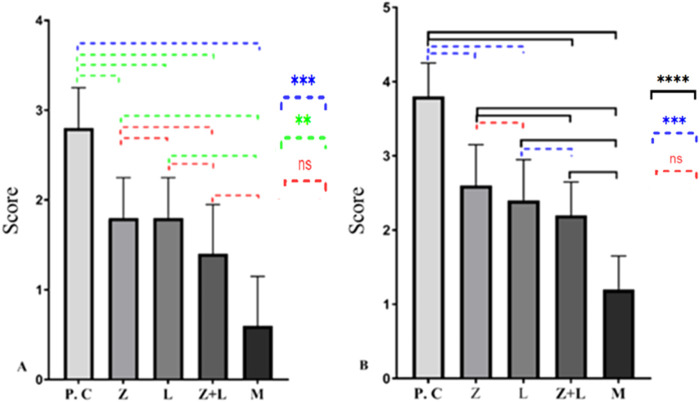
Macroscopic and microscopic condition of all treatments and control group. **(A)** Disease Activity Index. **(B)** Tissue damage. (^*^ indicates *p* < 0.05, ^**^ indicates *p* < 0.01, ^***^ indicates *p* < 0.001, ^****^ indicates *p* < 0.0001), (C.G: control group, L: L. casei*,* M: Mesalazine, L + M: L. casei + Mesalazine).

Figure 1A shows the disease activity index. The control group had the highest values, while the combined treatment group had the lowest. Significant statistical differences were observed between all treatment’s groups, except between the L. casei and mesalazine groups, and between the mesalazine and combined treatment groups.

Microscopic evaluation of slides (Figure 1B) show extensive mucosal damage in the control group, with over 90% necrotic and destroyed features in the colon’s epithelial layer. In healthy mice, normal tissue structure was observed, including the intestinal wall, crypts, muscular layer, mucous layer, Lieberkühn glands, submucosa, simple columnar epithelial tissue and connective tissue. In the untreated group, pathological changes were observed in the intestinal tissue structure, including disruption of the crypt structure and breakdown of the epithelial lining. Lieberkühn’s glands were somewhat undefined. Increased muscle tissue thickness and leukocyte infiltration were noted. There was a significant decrease in goblet cells, with signs of a repair in certain areas due to the prolonged process. The group that received L. casei showed slight improvement in intestinal tissue, with some presence of Lieberkühn glands and positive changes in goblet cells. The combined treatment group showed less tissue damage, with most parts of the intestine repaired, appearing somewhat similar to healthy mice.

### MPO and NO activity

**FIGURE 2:**
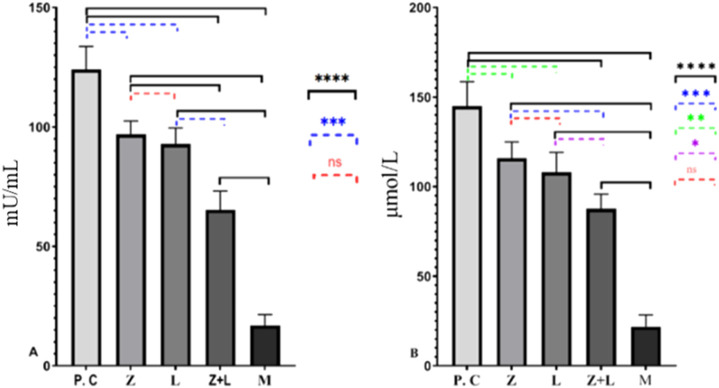
**(A)** Levels of MPO and **(B)** NO in the colon tissue of the study groups. In both figures, a significant statistical difference was seen between the studied groups. (^*^ indicates *p* < 0.05, ^**^ indicates *p* < 0.01, ^***^ indicates *p* < 0.001, ^****^ indicates *p* < 0.0001), (C.G: control group, L: L. casei, M: Mesalazine, L + M: L. casei + Mesalazine).

Based on the data presented in Figures 2A, B, the production of MPO and NO in the intestinal tissue of all treated mice was significantly reduced compared to the control group. Also, single-agent and two-agent treatment groups showed a statistically significant difference, and the two-agent treatment group showed the lowest levels of MPO and NO compared to all the studied groups.

### Cytokines

**FIGURE 3:**
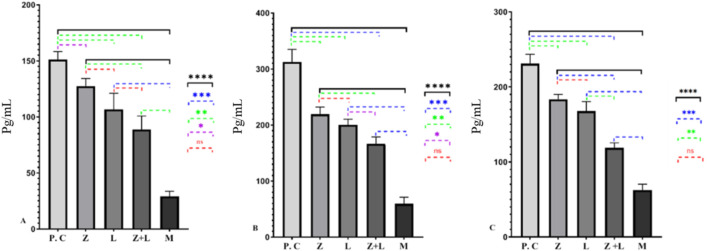
**(A)** The amount of IL-1ß, **(B)** The amount of IL-6, and **(C)** The amount of TNF-α cytokines production in different studied groups, for every group under study, a statistically significant difference was seen. (^*^ indicates *p* < 0.05, ^**^ indicates *p* < 0.01, ^***^ indicates *p* < 0.001, ^****^ indicates *p* < 0.0001), (C.G: control group, L: L. casei, M: Mesalazine, L + M: L. casei + Mesalazine).

According to Figure 3, in the untreated colitis group, the production of pro-inflammatory cytokines increased significantly compared to the single-factor and two-factor treatment groups. Also, a statistically significant difference was seen between the single-agent and two-agent treatment groups, and the lowest production values of inflammatory cytokines were observed in the two-agent treatment group.

### Cytokines, iNOS and, COX-2 genes expression

**FIGURE 4:**
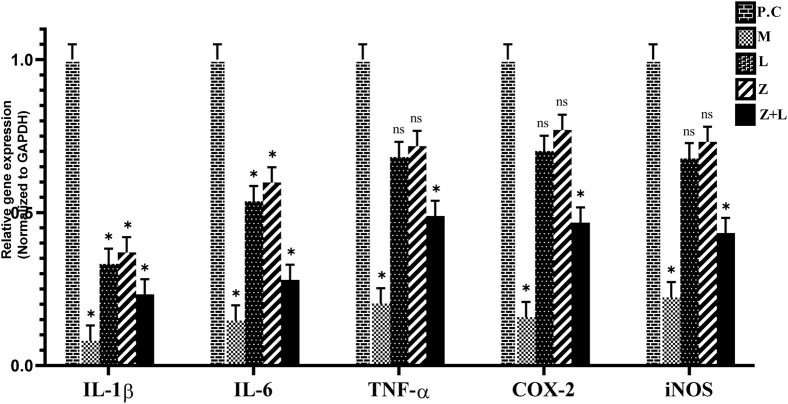
Relative expression diagram of IL-1ß, IL-6, TNF-α, COX-2, and iNOS genes in different study groups than the control group (C.G: control group, L: L. casei*,* M: Mesalazine, L + M: L. casei + Mesalazine).

The expression levels of the genes IL-1ß, IL-6, TNF-α, iNOS, and COX-2 were analyzed in different study groups using RT-PCR. The results presented in Figure 4 show a significant increase in these cytokines and genes in the control group. A significant reduction in expression levels was observed in all treatment groups compared to the control group. The lowest expression level was observed in the group treated with mesalazine. According to Figure 4, all treatment groups showed a statistically significant difference in the expression levels of IL-1ß and IL-6. However, in the L. casei treatment group, the expression levels of TNF-α, iNOS, and COX-2 did not show a statistically significant difference. Additionally, the expression levels of all genes in the groups receiving mesalazine and L. casei + mesalazine showed a significant reduction compared to the control group, And the expression levels of the genes in these two groups were approximately the same.

## Discussion

Animal models used for studying the immune response within the intestine have proven valuable in understanding IBD. This investigation specifically focuses on examining the effects of treatment with probiotic L. casei, mesalazine, and their combination on mice with UC. The findings showed that using L. casei probiotic and mesalazine reduced inflammation induced by colitis in mice. Additionally, it led to a significant decrease in disease activity index, the production of NO, MPO, inflammatory cytokine synthesis and expression, as well as COX-2 and iNOS genes. These results indicate that probiotic bacteria have the potential to reduce AA-induced damage (treatment was started after the onset of inflammation) (O’mahony et al., 2001). A study suggests that UC may be caused by gut microbiota. Clinical trials confirm that probiotics can help by changing the gut microbiota (Fedorak, 2009). One factor involved in managing and avoiding UC is the integrity of the intestinal mucosa. Known for their efficacy, probiotic strains like *Lactobacillus* and *Bifidobacterium* actively contribute to this stabilization (Saniabadi et al., 2014). Evidence supports the beneficial effects of probiotics, including their role in lowering cancer risk, functioning as antioxidants, and offering relief for UC (Argyri et al., 2013). Currently, probiotics are beneficial for autoimmune and digestive disorders (Sarowska et al., 2013). According to Je *et al.'s* research, probiotics can improve the function of the intestinal barrier and immune system as well as promote the survival of intestinal epithelial cells by inducing the generation of cytokines (Je et al., 2018). Evidence supports the widespread utilization of *Lactobacillus* as a supplement in foods owing to its advantageous impact on the host. These advantages encompass immune system modulation, anti-inflammatory traits, and antioxidant capabilities (Hu et al., 2020). In the study by Lin *et al.*, research confirms the antioxidant potential of *Lactobacillus*. Numerous studies have illustrated how components of *Lactobacillus* can mitigate the harm induced by oxidative stress, particularly in cases like UC (Lin and Chang, 2000). Several mechanisms have been identified. These actions encompass the modulation of gut microbiota, fortification of the function of the intestinal barrier, shielding the gastrointestinal epithelium from pathogenic intrusion, and bolstering immune functionality. In mice, oral treatment of L. casei ATCC 393 decreased intestinal damage from dextran sodium sulfate, according to research by Bellavia *et al.* In addition, research results show that the histological changes caused by TNBS are improved by oral consumption of L. casei (Bellavia et al., 2014). The L. casei probiotic utilized in Dong *et al.'s* study concurrently triggered the expression of both pro-inflammatory and anti-inflammatory cytokines (Dong et al., 2012). According to the survey by Hussein *et al.*, the consumption of probiotics reversed the massive architectural damage of the colon wall caused by acetic acid, which is evident by the restoration of the typical structure of the colon wall in H&E stained sections (Hussein et al., 2023). Some limitations in our study should be discussed. One of the limitations of the study was the high mortality of animals due to perforation of their intestines.

In 2008, Amany and Eman showed that the combination of the probiotic L. acidophilus with olsalazine resulted in significant improvement compared to using the probiotic or olsalazine as monotherapy. This improvement led to a reduction in diarrhea and rectal bleeding, along with a significant decrease in the Disease Activity Index (DAI) and inflammatory markers (Abdin and Saeid, 2008). Additionally, Tursi and colleagues in 2004 demonstrated that the combination of balsalazide and VSL#3 is a very good choice for treating active ulcerative colitis, instead of using balsalazide alone or mesalazine (Tursi et al., 2004). In 2010, Tursi and colleagues found that VSL#3 supplementation after 8 weeks was able to reduce disease activity scores in patients affected by relapsing mild to moderate UC who were concurrently under treatment with 5-ASA and/or immunosuppressants (Tursi et al., 2010). In the study by Palumbo *et al.*, all UC patients treated with combination therapy (mesalazine + Ligilactobacillus salivarius, L. acidophilus, and Bifidobacterium bifidus BGN4) showed improvement in the Disease Activity Index and reduced stool frequency, with endoscopic pictures showing improvement in gut mucosa signs compared to controls who received only mesalazine. These findings are consistent with our results, indicating that combination therapy improves clinical outcomes (Palumbo et al., 2016). Tursi et al. (2013) discovered that administering mesalazine and *Lactobacillus* casei concurrently proved to be more successful than a placebo in enhancing the recovery of patients experiencing symptomatic and uncomplicated diverticular disease (Tursi et al., 2013). Our study highlights the potential of Combined treatment with mesalazine and L. casei as a promising approach for UC. Using a model induced by acetic acid, we observed the effectiveness of L. casei and mesalazine in alleviating inflammation and shielding the intestinal tissue from damage. Notably, a substantial reduction in crucial inflammatory markers, including NO, MPO, IL-1β, IL-6, and TNF-α, were evident after treatment with L. casei and mesalazine. Additionally, the downregulation of pro-inflammatory genes such as IL-1β, IL-6, TNF-α, iNOS, and COX-2 further supports its therapeutic potential. These findings suggest the simultaneous use of L. casei and mesalazine is an effective treatment for managing this inflammatory condition.

However, additional research is imperative to explore its therapeutic advantages, safety profile, and long-term implications.

## Data Availability

The raw data supporting the conclusions of this article will be made available by the authors, without undue reservation.
